# Genomic and environmental influences on resilience in a cold‐water fish near the edge of its range

**DOI:** 10.1111/eva.13313

**Published:** 2021-11-09

**Authors:** Amanda S. Ackiss, Madeline R. Magee, Greg G. Sass, Keith Turnquist, Peter B. McIntyre, Wesley A. Larson

**Affiliations:** ^1^ Wisconsin Cooperative Fishery Research Unit College of Natural Resources University of Wisconsin‐Stevens Point Stevens Point Wisconsin USA; ^2^ U.S. Geological Survey Great Lakes Science Center Ann Arbor Michigan USA; ^3^ Wisconsin Department of Natural Resources Madison Wisconsin USA; ^4^ Escanaba Lake Research Station Wisconsin Department of Natural Resources Boulder Junction Wisconsin USA; ^5^ Department of Natural Resources and the Environment Cornell University Ithaca New York USA; ^6^ U.S. Geological Survey Wisconsin Cooperative Fishery Research Unit College of Natural Resources University of Wisconsin‐Stevens Point Stevens Point Wisconsin USA; ^7^ National Oceanographic and Atmospheric Administration National Marine Fisheries Service Alaska Fisheries Science Center Auke Bay Laboratories Juneau Alaska USA

**Keywords:** deleterious mutations, genetic drift, major histocompatibility complex, marginal populations, mutational load, small populations

## Abstract

Small, isolated populations present a challenge for conservation. The dueling effects of selection and drift in a limited pool of genetic diversity make the responses of small populations to environmental perturbations erratic and difficult to predict. This is particularly true at the edge of a species range, where populations often persist at the limits of their environmental tolerances. Populations of cisco, *Coregonus artedi*, in inland lakes have experienced numerous extirpations along the southern edge of their range in recent decades, which are thought to result from environmental degradation and loss of cold, well‐oxygenated habitat as lakes warm. Yet, cisco extirpations do not show a clear latitudinal pattern, suggesting that local environmental factors and potentially local adaptation may influence resilience. Here, we used genomic tools to investigate the nature of this pattern of resilience. We used restriction site‐associated DNA capture (Rapture) sequencing to survey genomic diversity and differentiation in southern inland lake cisco populations and compared the frequency of deleterious mutations that potentially influence fitness across lakes. We also examined haplotype diversity in a region of the major histocompatibility complex involved in stress and immune system response. We correlated these metrics to spatial and environmental factors including latitude, lake size, and measures of oxythermal habitat and found significant relationships between genetic metrics and broad and local factors. High levels of genetic differentiation among populations were punctuated by a phylogeographic break and residual patterns of isolation‐by‐distance. Although the prevalence of deleterious mutations and inbreeding coefficients was significantly correlated with latitude, neutral and non‐neutral genetic diversity were most strongly correlated with lake surface area. Notably, differences among lakes in the availability of estimated oxythermal habitat left no clear population genomic signature. Our results shed light on the complex dynamics influencing these isolated populations and provide valuable information for their conservation.

## 1 INTRODUCTION

Understanding the nature of resilience and the capacity for evolution in the face of rapidly changing environments is a cornerstone of conservation genomics. Sources of selective pressure are numerous, and studies using genotype–environment association analyses that detect locally adapted populations can provide valuable insight on the environmental forces that influence population viability (Berg et al., 2015; Eizaguirre et al., 2012; Yeaman et al., 2016). Since heritable genetic variation provides the foundation for adaptive capacity under selection, the maintenance of diversity is fundamental to the preservation of healthy populations (Hoban et al., 2013; Sgrò et al., 2011). The mutations that underlie allelic diversity occur on a spectrum that can be broadly reduced to three categories (advantageous, neutral, or deleterious), with the frequency of these categories varying under mutation‐selection balance.

In small populations, the mutation‐selection balance can often be upset, making the frequency of deleterious mutations a conservation concern. Most mutations are mildly deleterious (Fay et al., 2001; Keightley & Lynch, 2003; Ohta, 1995), and in a large, randomly outcrossing population, recombination and segregation are expected to keep the mutational load from significantly affecting absolute mean fitness (Agrawal & Whitlock, 2012; Haag & Roze, 2007). The role of genetic drift increases as population sizes decline, such that natural selection may be ineffective at removing deleterious mutations (Whitlock & Bürger, 2004; Wright, 1931), leading to “mutational meltdown” and eventually extirpation of small populations (Lynch et al., 1995). As new genomic tools have become available to identify putatively deleterious variants, studies of small populations are quantifying deleterious mutations to assess the viability of at‐risk populations (Andrews, 2010) and to inform conservation efforts (e.g., Ferchaud et al., 2018; Grossen et al., 2020; Perrier et al., 2017).

Conservation of small populations is inherently challenging, especially when they are isolated from nearby populations (Pekkala et al., 2012; Ralls et al., 2018). Without the option of being rescued by immigration, isolated populations are especially susceptible to environmental fluctuations and local habitat degradation (Pimm, 1991). These unique attributes mean that conservation principles developed for larger populations or metapopulations may not be suitable for managing small populations. For example, many conservation plans attempt to understand how species will respond to rising global temperatures on relatively large spatial scales (e.g., 100s of kms), but the viability of isolated, small populations depends on the details of how their local environment changes—which may deviate from large‐scale trends. For example, aquatic ecosystems vary widely in the source of water (Lisi et al., 2013), habitat dimensions and nutrient loads (Olden et al., 2001), and precipitation inputs (Bay et al., 2018) even within the same geographic region. Additionally, environmental variation at local scales has been linked to immunocompetence in aquatic populations (Larson et al., 2016). In habitats separated by as little as 1 km, Larson et al. (2016) found differences in water temperature were significantly correlated with diversity in a major histocompatibility complex (MHC) gene involved in immune system response. Understanding how these local factors influence the genetic integrity of small, isolated populations is vital for managing them.

Here, we use cisco (*Coregonus artedi*) as a model organism to understand how spatial and environmental factors influence genetic integrity of small, isolated populations under a changing climate. Cisco are an important forage fish found in deep, cold lakes across northern North America (Scott & Crossman, 1973). As cold‐water specialists, ciscoes require both cold‐water temperatures and sufficient dissolved oxygen, found within the oxythermal habitat layer, to survive the stratification that occurs in deep lakes during the summer (Cahn, 1927; Frey, 1955; Jacobson et al., 2008; Lyons et al., 2018). Rising global temperatures have been linked to freshwater fish die‐offs (Till et al., 2019), and declines in abundance as well as complete extirpations of inland lake cisco populations have been documented along the southern edge of the species range in Minnesota, Wisconsin, and Indiana (Honsey et al., 2016; Jacobson et al., 2012). Contemporary surveys across 133 Wisconsin lakes that historically contained ciscoes reveal a mosaic pattern of extirpation rather than a clear latitudinal gradient (Lyons et al., 2015); geographic proximity does not predict which populations were extirpated and which continue to thrive, suggesting that local factors mediate population outcomes.

A growing body of research focuses on using oxythermal habitat modeling to predict extirpations of cold‐water fish populations and design conservation strategies (Jacobson et al., 2013; Jiang et al., 2012; Lyons et al., 2018; Magee et al., 2019; Renik et al., 2020), but this perspective has not been fully integrated with genomic analyses of cisco. Previous genetic analyses across the cisco species range using mitochondrial and microsatellite DNA have found evidence for at least two major lineages (Bernatchez & Dodson, 1990; Turgeon & Bernatchez, 2001a, 2001b, 2003). More recently, a study of cisco in inland lakes of Ontario using a genomic method similar to restriction site‐associated DNA (RAD) sequencing detected two lineages and measured high levels of differentiation among lake systems (Piette‐Lauzière et al., 2019), but did not quantify genomic diversity within or among lakes. The southern edge of the species’ range offers opportunities to assess how genetic metrics correlate with both spatial variables and static and climate‐dynamic environmental variables that may be associated with population persistence.

In this study, we applied multiple genomic tools to profile surviving populations of cisco near their southern range limit in Minnesota, Wisconsin, Indiana, and Michigan, and reconstructed the dynamics of oxythermal habitat availability in each lake across a 37‐year period before genetic sampling. We used restriction site‐associated DNA capture (Rapture) sequencing Ali et al. (2016) to survey genomic diversity and differentiation, and to enumerate the presence and frequency of putative deleterious mutations among lakes. We also amplified the MHC IIβ exon 2 to examine the contributions of neutral or pathogen‐mediated adaptive processes to haplotype diversity across these southern inland cisco populations. To understand the spatial and environmental context for genomic variation in ciscoes, we correlate each of these genetic metrics to the latitude, size, depth, and oxythermal habitat availability in each lake.

## 2 METHODS

### 2.1 Tissue sampling and DNA extraction

**FIGURE 1:**
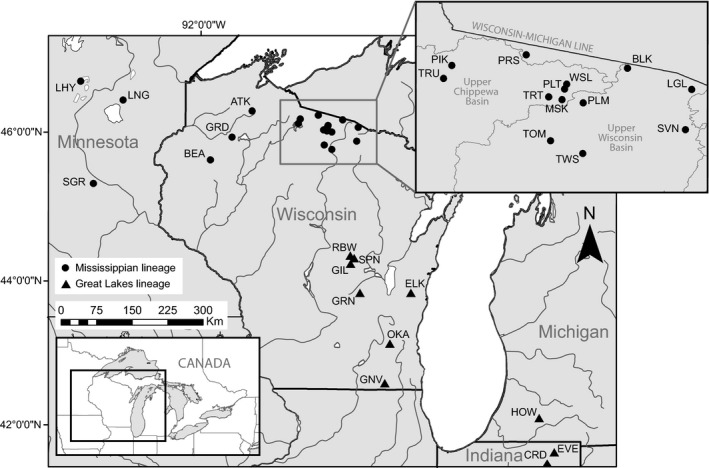
Map showing the 29 sampled cisco lakes. Inset (bottom left): box indicates the location of the sampling area within the Laurentian Great Lakes region. Inset (top right): sampled lakes in northern Wisconsin. Lake names and site data can be found with site codes in Table 1. We detected two phylogenetic lineages in our sampling area. Populations associated with the Mississippian (MS) lineage are denoted with circles and populations associated with the Great Lakes (GL) lineage are denoted with triangles

Cisco used in our study were collected from 29 lakes across the southern part of the species’ range using vertical gill nets or from die‐offs during 2012–2019 (Figure 1; Table 1). To our knowledge, all sampled lakes contained the standard inland cisco ecotype except for one lake, Atkins (ATK), where the cisco population is comprised of a dwarf cisco ecotype. Fin clips were provided by Michigan State University (Howard Lake), the Indiana Department of Natural Resources and Purdue University (Indiana lakes), the Wisconsin Department of Natural Resources (Wisconsin lakes), and the Minnesota Department of Natural Resources and the University of Minnesota‐Duluth (Minnesota lakes). DNA from fin tissue was extracted using Qiagen DNeasy^®^ Blood & Tissue Kit reagents.

**TABLE 1:** Location and environmental data for the 29 cisco lakes sampled across the southern periphery of the species range. Complete oxythermal data can be found in Data S2

State	Lake	Site code	Latitude	Longitude	Max Depth (m)	Surface Area (km^2^)	TDO3_max_
Minnesota	Lower Hay	LHY	46.6693	−94.2843	42.06	57.75	22.5516
	Sugar	SGR	45.3189	−94.0418	21.03	4.1	22.9099
	Long	LNG	46.4895	−93.4938	35.36	1.76	17.4888
Wisconsin	Bear	BEA	45.6343	−91.8248	27	5.5	20.4916
	Grindstone	GRD	45.9353	−91.4154	18	12.6	22.7022
	Atkins	ATK	46.2789	−91.0367	24	0.7	12.5363
	Trude	TRU	46.1148	−90.1579	15	54.8	22.1987
	Pike	PIK	46.1747	−90.1196	25	0.8	11.4723
	Presque Isle	PRS	46.2227	−89.7798	24	5.2	17.8400
	Trout	TRT	46.0303	−89.6766	36	15.4	11.4034
	White Sand	WSL	46.0887	−89.5941	2	3	12.1445
	Big Muskellunge	MSK	46.0174	−89.6149	21	3.8	16.1785
	Pallette	PLT	46.0665	−89.6039	20	0.7	15.2114
	Plum	PLM	46.0033	−89.5193	17	4.5	22.2666
	Tomahawk	TOM	45.8304	−89.6675	26	14.4	14.8864
	Two Sisters	TWS	45.7716	−89.5207	19	2.9	13.3695
	Black Oak	BLK	46.1612	−89.3155	26	2.4	9.4554
	Long	LGL	46.0645	−89.0225	29	3.5	12.5157
	Sevenmile	SVN	45.8811	−89.0506	13	2	22.3921
	Rainbow	RBW	44.3411	−89.1504	29	2.4	10.6171
	Spencer	SPN	44.2897	−89.1043	16	0.3	12.5953
	Gilbert	GIL	44.2120	−89.1675	20	0.6	12.3836
	Green	GRN	43.8186	−88.9876	72	29.7	13.5789
	Elkhart	ELK	43.8262	−88.0240	36	1.2	11.5196
	Okauchee	OKA	43.1262	−88.4232	29	4.8	20.5535
	Geneva	GNV	42.5637	−88.5179	41	21.3	11.2306
Michigan	Howard	HOW	42.0808	−85.5889	16.15	1.84	14.8317
Indiana	Eve	EVE	41.5603	−85.3199	12.8	0.3	17.4626
	Crooked	CRD	41.2634	−85.4803	32.9	1.6	13.2668

### 2.2 Development of Rapture panel

We developed a Rapture panel using restriction site‐associated DNA (RAD) data from 129 individuals (data previously published in Euclide et al., 2020) to increase genotyping efficiency and the number of individuals that could be multiplexed on each sequencing lane. The 129 individuals were sampled from multiple populations in Minnesota (*N* = 24), northern Wisconsin (*N* = 37), central Wisconsin (*N* = 26), southern Wisconsin (*N* = 10), Indiana (*N* = 13), and Michigan (*N* = 19). RAD library preparation was conducted using the *SbfI* enzyme according to the methods of Ali et al. (2016), and libraries were sequenced on two lanes of an Illumina HiSeq 4000 at the Michigan State Genomics Core Facility. Sequence data were analyzed with Stacks v1.46 (Catchen et al., 2011, 2013) with the following parameters: *process_radtags* (flags = c, ‐q, ‐r, ‐t 140), *ustacks* (flags = ‐m 3, ‐M 5, ‐H ‐‐max_locus_stacks 3, ‐‐model_type bounded, ‐‐bound_high 0.05), *cstacks* (‐n of 4, 29 individuals with the most data included in the catalog), and *populations* (flags = ‐r 0.7, ‐‐min_maf 0.05). In addition, since all salmonids including cisco have experienced a recent genome duplication, putatively paralogous loci were identified with the program HDPlot (McKinney et al., 2017), and any loci with heterozygosity >0.6 or a read ratio deviation >10 and <–6 were classified as duplicated. We chose a total of 11,330 tags containing high‐quality singleton SNPs (i.e., not from duplicated regions of the salmonid genome) and 1670 tags containing duplicated SNPs for a total of 13,000 tags. Sequence data for the loci that met our quality standards were sent to Arbor Biosciences (Ann Arbor, MI) for Rapture bait development. Arbor Biosciences conducted additional quality filters to ensure robust performance and synthesized two baits per‐locus when possible, resulting in a final panel of 7753 loci and 14,382 unique baits.

### 2.3 Rapture loci processing

Rapture libraries were prepared with the RAD protocol used for panel development followed by bait capture using the myBaits protocol v4.01 (Arbor Biosciences) with minor modifications. Libraries were hybridized with the bait mixture for 16 hr at 65°C and amplified with 10 PCR cycles, universal primers, and an annealing temperature of 56°C. Baited Rapture libraries were purified with 1X AMPureXP beads and sequenced on five Illumina HiSeq 4000 lanes at either the Michigan State Genomics Core Facility or Novogene.

Raw sequences were processed with Stacks v2.41 (Rochette et al., 2019). Sequences were demultiplexed by barcode, filtered for presence of the enzyme cut‐site and quality, and trimmed in the subprogram *process_radtags* (parameter flags: ‐e *SbfI* ‐c ‐q ‐r ‐t 140 ‐‐filter_illumina ‐‐bestrad). Filtered reads for each individual were aligned to create matching stacks with *ustacks* (flags: ‐‐disable‐gapped ‐m 3 ‐M 5 ‐H ‐‐max_locus_stacks 4 ‐‐model_type bounded ‐‐bound_high 0.05). A master catalog of consensus loci was built in *cstacks* from a catalog of 51 *C*. *artedi* used in the development of a cisco linkage map (Blumstein et al., 2020) that was appended with five individuals from each lake that retained the highest number of reads after *process_radtags* (flags: ‐n 3 ‐p 6 –disable_gapped). Locus stacks for individuals were matched to the catalog using *sstacks* (flag: ‐‐disable_gapped), data were oriented by locus in *tsv2bam*, and reads were aligned to loci and SNPs were called with *gstacks*. SNPs genotyped in >30% of individuals (flag: ‐r 0.3) were exported with *populations* to a variant call format (vcf) file.

SNP filtering was performed with vcftools v0.1.15 (Danecek et al., 2011) and included the following: (1) removing loci genotyped in <70% of individuals; (2) removing individuals missing >50% of loci; and (3) removing loci with a minor allele frequency <0.01. Any loci remaining after primary filtering that were not genotyped in every lake were also removed. Putatively paralogous loci were identified with the program HDPlot (McKinney et al., 2017), and loci with heterozygosity >0.5, a read ratio deviation >15 and < –15, and an allele balance >0.7 and <0.3 were removed. Finally, loci on the same RAD tag are likely to be physically linked. Therefore, only the SNP with the highest minor allele frequency on each tag was included in the final dataset. File format conversions were performed with PGDSpider v2.1.1.5 (Lischer & Excoffier, 2011).

### 2.4 Genomic differentiation and diversity

We calculated diversity statistics for each population with GenoDive v3.0 (Meirmans, 2020), including observed and expected heterozygosity, an inbreeding coefficient (*G*_IS_, a heterozygosity‐based analog of *F*_IS_; Nei, 1987), and the proportion of polymorphic alleles present in each population. Effective population size (*N*_e_) was estimated in each population with the bias‐corrected linkage disequilibrium method (LDNE; Hill, 1981; Waples, 2006; Waples & Do, 2010) in the software package NeEstimator v2.1 (Do et al., 2014). We restricted pairs of loci used in calculations to those comprised of two loci on different linkage groups of the cisco linkage map (Blumstein et al., 2020) using the LD locus pairing option to correct for the effects of physical linkage on the estimates of *N*_e_ (Waples et al., 2016). We used a p‐crit of 0.05 (Waples et al., 2016) to reduce potential bias introduced by low frequency alleles. *N*_e_ calculations using the linkage disequilibrium method can be biased slightly downward when individuals from multiple cohorts are included in the sample due to a slight Wahlund effect (7% downward bias on average; Waples et al., 2014). Nonetheless, this small bias should not greatly affect the interpretation of the *N*_e_ results.

We also used GenoDive to estimate standardized pairwise genetic differentiation between populations (*F*_ST_). Analysis of genetic differentiation across our populations indicated that we likely sampled inland lakes across a putative phylogeographic break. We constructed a population tree from allele frequency data using the software POPTREE2 to identify which populations belonged to which divergent clade. Genetic distance between inland lake populations was measured with *D_A_* (Nei et al., 1983), and trees were generated using the neighbor‐joining (NJ) method of Saitou and Nei (1987) with 1000 bootstrap replicates. Correlation between Slatkin's linearized *F*_ST_ (*F*_ST_ /(1 − *F*_ST_)) (Rousset, 1997) and planar geographic distance between populations was examined using Mantel and partial Mantel tests to test for isolation‐by‐distance (IBD) while accounting for lineage effects (Mantel, 1967; Meirmans, 2012; Sokal, 1979), and the regression between *F*_ST_/1 − *F*_ST_ and geographic distance (km) was plotted. We also examined the relationship of genetic distance and spatial heterogeneity in effective population size by testing the correlation between pairwise genetic differentiation (*F*_ST_ /(1 − *F*_ST_)) and the *di* metric of Prunier et al. (*distance based on the inverse*; 2017) with log (surface area) as a proxy for local carrying capacity, as well as the correlation of average pairwise genetic differentiation to log (surface area) as implemented in Perrier et al. (2017). Finally, we used analysis of molecular variance (AMOVA) in GenoDive to test for the relative amounts of variance in our dataset that could be explained by individual, population, or lineage effects.

### 2.5 Identifying putatively deleterious mutations

We identified putative deleterious mutations in our Rapture data using the program PROVEAN (Choi et al., 2012) and methods similar to Perrier et al. (2017) and Ferchaud et al. (2020), Ferchaud et al. (2018); scripts included with our supplementary material on Dryad). First, we created a fasta file including each allele for each locus from the catalog.fa file output from Stacks and a VCF file using the script make_fasta_with_all_alleles.py. We then aligned sequences for each RAD locus to the protein coding sequence from the Atlantic salmon (*Salmo salar*) genome (Lien et al., 2016) using BLASTX. Hits with a sequence overlap of >40 amino acids and a percent identity >90% were retained, and if more than one hit for a given locus met these parameters, the hit with the highest % identity was kept (parse_blast_results_best_align_delet_mut_consensus_seqs.py). Next, portions of sequences for each locus that aligned to Atlantic salmon proteins were translated into protein coding sequences with each allele representing a unique sequence (pull_seqs_to_translate_consensus_blast.py). Protein sequences for loci with nonsynonymous mutations were then converted to PROVEAN input files (generate_provean_files.py). We then used PROVEAN linked to the NCBI BLAST database v4 (https://ftp.ncbi.nlm.nih.gov/blast/db/v4/, author note: v5 was available but incompatible with PROVEAN at the time of analysis) to assess whether each mutation was putatively deleterious (PROVEAN score ≤ −2.5) and outputs were parsed with a custom script (parse_provean_output.py). We interpreted the allele that generated the negative PROVEAN score to be the deleterious allele and the alternative allele (≥2.5) to be neutral, following similar approaches in the literature (e.g., Conte et al., 2017). Finally, we calculated two metrics to assess the frequency of deleterious mutations in each population: (1) average allele frequency of deleterious alleles (AF_del mut_), and (2) proportion of deleterious mutations (Prop_del mut_), which was calculated as the number of loci with an allele frequency >0 for a deleterious mutation in that population divided by the total number of deleterious mutations in the dataset.

### 2.6 MHC processing

We amplified a 300‐bp region of the MHC IIβ exon 2 using primers and reaction conditions described in Pavey et al. (2011). We then attached sequencing barcodes and adapters and normalized the DNA using the genotyping‐in‐thousands by sequencing (GT‐seq) protocol (Campbell et al., 2015) following Bootsma, Gruenthal, et al. (2020). Each plate was barcoded and then batch normalized using SequalPrep DNA Normalization plates (Invitrogen). Normalized products were pooled, then cleaned and size selected using AMPureXP magnetic beads (Beckman Coulter, Brea, CA). Products were run on an agarose gel to verify size ranges and quantified using a Qubit 2.0 fluorometer. Amplicons were sequenced on an Illumina MiSeq 2x300 flowcell at the University of Wisconsin Biotechnology Center in Madison, Wisconsin.

After sequencing, terminal read quality was examined with FastQC (Andrews, 2010), and sequences were trimmed to 176 bp with the command line tool fastx_trimmer from FASTX‐Toolkit (https://github.com/agordon/fastx_toolkit). Forward and reverse reads were merged using the software tool FLASH (‐m 50, ‐M 150; Magoč & Salzberg, 2011), and the final 300 bp reads were quality filtered with the perl tool PRINSEQ‐lite (http://prinseq.sourceforge.net/) using a minimum base quality score (min_qual_score) of 30. The demultiplexing process removed barcodes from sequences so those were extracted from sequence headers and inserted at the beginning of reads (barcodes‐min.py) before importing into the software jMHC (Stuglik et al., 2011). To maximize the number of sequences imported to the jMHC SQLite database, the forward and reverse primers were truncated to 10 bps. Allowing for one mismatch per 12 bp i7+i5 barcode, reads were then searched from the forward orientation, and if both primers and barcode were found, the read was imported to the database. Unique haplotypes with >50 reads were then exported for analysis. An MHC haplotype was present in an individual if the haplotype contained >10% of reads in that individual. MHC haplotypes that were only found once in the dataset were likely errors and were excluded. Many individuals contained >2 MHC haplotypes, indicating that our primers amplified multiple MHC genes, which is an expected result for salmonids (Harstad et al., 2008). However, we were unable to assign dosage (i.e., copy number) for each allele because there were no clear differences in read count distributions among putative genotype classes. Our genotype matrix is therefore coded as presence/absence data rather than complete genotype calls.

We used the generated MHC genotype matrix to investigate diversity, selection, and differences in MHC haplotype frequencies among populations. Estimates of allelic diversity can be highly influenced by sample sizes, but rarefaction methods such as that implemented in the program HPRARE (Kalinowski, 2005) can be used to estimate unbiased allelic richness. Unfortunately, this program can only take diploid data and many of the individuals in our dataset contained >2 MHC alleles. We therefore calculated a rarefied average number of MHC haplotypes present in each population (*N*_hap_) by randomly sampling *N* = 5 individuals (minimum sample size in our study) from each population 1000 times and calculating the mean number of haplotypes found in those samples. We also estimated the proportion of individuals in each population that contain more than one allele (Prop_poly_) and the proportion of individuals in a population with more than two MHC alleles (Prop_>2 alleles_). We conducted a codon‐based Z‐test of selection in MEGA 7 using the Nei‐Gojobori method (Nei & Gojobori, 1986) to test whether the ratio of nonsynonymous mutations (DN) was greater than synonymous mutations (DS). Finally, we visualized MHC haplotype frequencies calculated as the number of individuals containing a given haplotype divided by the total number of individuals for each population with a stacked bar plot. This metric was standardized to 1 by dividing by the sum of the frequencies for each population which was >1 as individuals can have >1 haplotype.

### 2.7 Oxythermal habitat modeling

We used the open source, vertical one‐dimensional hydrodynamic General Lake Model (GLM; Hipsey et al., 2019) v.2.1.8, coupled with the Aquatic EcoDynamics (AED2) model (Hipsey et al., 2020) to develop daily water temperature and dissolved oxygen profiles for each lake over the years 1979–2015. GLM and AED are available online (https://github.com/AquaticEcoDynamics/GLM and https://github.com/AquaticEcoDynamics/libaed2). This model simplifies lakes using a vertical Lagrangian layer approach, where horizontal variability is ignored, and each layer's thickness dynamically changes in response to water density (Hipsey et al., 2019). The water quality module, AED2, was configured to simulate the dynamics of dissolved oxygen, carbon, silica, nitrogen, phosphorus, organic matter, phytoplankton (three functional groups representing cyanobacteria, diatoms, and chlorophytes), and zooplankton (two functional groups representing copepods and *Daphnia*).

Historic climate drivers for each lake were estimated using data from (Winslow et al., 2017) and expanded following similar methods for lakes that were not available in the original dataset. The model does not consider surface inflow of water into the system, and we assumed that water levels remained constant over the study period. To counteract any long‐term negative change in lake level, we applied a small correction to precipitation in summer as in Winslow et al. (2017). *In situ* water temperature and dissolved oxygen profiles were collected from the NTL‐LTER program (https://lter.limnology.wisc.edu/), US Geological Service, Michigan DNR, Minnesota DNR, Wisconsin DNR, University of Wisconsin‐Madison, and Purdue University and compiled as supplemental material on Dryad. We performed a manual calibration for each lake based on the full set of temperature and dissolved oxygen profiles available. Model parameters were first calibrated for water temperature and second for dissolved oxygen. Lake‐specific data were only available for water temperature and dissolved oxygen, so default values were used for other model parameters, as is standard practice. Models were run at an hourly timestep starting April 4, 1979, and continuing through December 31, 2015, with output profiles once daily at noon. Model configuration files, input, and output profiles are accessible on Dryad.

### 2.8 Environmental variables in sampled cisco populations

We obtained data on surface area and maximum depth from Minnesota Department of Natural Resources databases for Minnesota lakes, from Renik et al. (2020) for Wisconsin Lakes, from Honsey (2014) for Indiana lakes, and from an EPA report for Howard Lake in Michigan (EPA, 1975). Environmental variables to estimate cisco oxythermal habitat from the hydrodynamic model were as follows: maximum daily TDO3 (temperature at 3 mg L^−1^ dissolved oxygen; Jacobson et al., 2010); seven‐day maximum TDO3 (TDO3_7day_); 30‐day maximum TDO3 (TDO3_30day_); COSD (Cumulative Oxythermal Stress Dosage; Magee et al., 2019); minimum yearly oxythermal habitat thickness; and minimum yearly oxythermal habitat volume estimated from model‐derived daily water temperature and dissolved oxygen profiles from 1979 to 2015. Maximum daily TDO3 (TDO3_max_) values were calculated following Jacobsen et al. (2010), and TDO3_7day_ and TDO3_30day_ values were calculated following (Jiang et al., 2012) using either a 7‐ or 30‐day moving average. COSD, which considers both the magnitude and duration of oxythermal stress conditions, was calculated as in Magee et al. (2019) using 3 mg L^−1^ DO and 17°C thresholds. Daily vertical habitat thickness was calculated from profiles by subtracting the depth at which the thermal profile reached 17°C from the depth at which the DO profile reached 3 mg L^−1^. The minimum yearly vertical habitat thickness was determined to be the minimum daily vertical habitat thickness value during the open‐water period and could be negative in instances where 3 mg L^−1^ of DO was achieved in the metalimnion or epilimnion. The minimum yearly oxythermal habitat volume was estimated by multiplying the minimum yearly vertical thickness by the lake's surface area. Calculations were performed using Matlab R2019a, and code is available as supplemental material on Dryad. One value for each environmental variable was calculated for each study year, and we also extracted long‐term averages and extreme values that could lead to bottlenecks in habitat availability. Lake surface area and maximum depth were considered as static descriptors of each lake.

### 2.9 Correlating genetic variables with spatial and environmental parameters

We screened spatial and environmental variables for multicollinearity using pairwise correlations and variance inflation factors (VIF) with the R packages Hmisc (Harrell Jr & others, 2020) and usdm (Naimi et al., 2014). Predictors were pruned iteratively based on a threshold of VIF <5 and pairwise Pearson's correlation coefficients of |*r*| >0.7 (Dormann et al., 2013). We conducted RDA analysis in the R package vegan (Okansen et al., 2016) to test for a relationship between spatial and environmental variables and genetic variables. For this analysis, the response matrix was composed of genetic diversity metrics and the predictor matrix was composed of spatial and environmental variables. Our choice to conduct RDA instead of canonical correspondence analysis, a similar multivariate method, was informed by a detrended correspondence analysis, which indicated that variables were linear and thus more appropriate for RDA (Legendre & Legendre, 1998). The significance of each predictor (i.e., their influence on variation in genetic metrics) was assessed with ANOVAs. We then used multiple linear regressions to test for relationships between genetic (response) variables and environmental (predictor) variables. The relative importance of each variable was assessed with the lmg method implemented in the R package relaimpo (Groemping, 2006). Finally, we conducted univariate linear regressions with the ‘lm’ function in R (and visualized with ggplot2; Wickham, 2011) to test for individual relationships between genetic variables and spatial and environmental variables that were significant in the RDA.

## 3 RESULTS

### 3.1 Genomic differentiation and diversity with Rapture loci

Sequence reads were generated for a total of 850 samples from our 29 lakes, and after processing in Stacks, 543,108 shared variant sites were genotyped in >30% of individuals. A total of 59,865 loci were genotyped in >70% of individuals. Eighty individuals were dropped for missingness >50%. A large percentage of samples dropped were from previously archived DNA extracts, dried fin clips, and/or collections from die‐offs. The loss of these individuals was most likely due to low quality (degraded) DNA in the extracts used for Rapture library preparation, which has been shown to highly impact the percentage of retained tags (Graham et al., 2015). The dataset contained 16,584 loci after filtering for minor allele frequency. Following the removal of putatively paralogous loci (*n* = 3578), removal of loci that were not genotyped in every lake (*n* = 59), and filtering to a single SNP per Rapture locus, 7546 SNPs genotyped in 770 individuals (see Table 2 for number of individuals genotyped per population) remained for analysis.

**TABLE 2:** Rapture and MHC data for sampled lakes

Genetic lineage	Site code	Rapture data								MHC data			
		*N*	*H*_o_	*H*_e_	*G*_IS_	Prop_poly_	*N*_e_ (CI)	AF_del mut_	Prop_del mut_	*N*	N_haps_	Prop_poly_	Prop_>2 alleles_
Mississippian (MS)	LHY	26	0.236	0.273	0.136	0.917	561 (504–631)	0.72	1	25	7.96	0.96	0.24
	SGR	23	0.125	0.173	0.276	0.781	36 (35–37)	0.71	0.97	18	7.43	0.78	0.50
	LNG	18	0.177	0.254	0.302	0.866	437 (362–552)	0.73	1	11	6.41	0.82	0.36
	BEA	30	0.130	0.178	0.267	0.783	519 (456–602)	0.70	0.93	26	5.45	0.69	0.46
	GRD	28	0.170	0.224	0.244	0.854	207 (198–217)	0.70	0.97	27	5.37	0.78	0.33
	ATK	54	0.103	0.125	0.173	0.706	128 (124–132)	0.74	0.89	41	2.95	0.90	0.34
	TRU	30	0.257	0.264	0.028	0.897	131 (128–134)	0.71	0.99	5	8.00	0.80	0.40
	PIK	30	0.185	0.216	0.142	0.826	584 (521–665)	0.73	0.96	21	2.68	0.48	0
	PRS	30	0.178	0.245	0.273	0.880	155 (151–161)	0.73	0.98	27	5.25	0.67	0.33
	TRT	13	0.174	0.243	0.286	0.851	231 (201–271)	0.71	0.97	15	8.29	1.00	0.47
	WSL	30	0.228	0.237	0.039	0.868	1781 (1355–2593)	0.70	0.97	28	5.64	0.79	0.39
	MSK	29	0.154	0.209	0.263	0.819	157 (151–164)	0.73	0.95	24	4.36	0.83	0
	PLT	30	0.176	0.192	0.084	0.809	798 (678–970)	0.73	0.95	28	3.27	0.61	0
	PLM	30	0.211	0.221	0.046	0.845	636 (567–726)	0.74	0.96	26	5.91	0.73	0.15
	TOM	30	0.215	0.257	0.163	0.902	532 (486–587)	0.74	0.98	26	8.28	0.96	0.38
	TWS	30	0.202	0.219	0.079	0.849	261 (248–275)	0.74	0.98	29	5.37	0.90	0.38
	BLK	24	0.127	0.190	0.330	0.794	63 (62–65)	0.74	0.94	14	4.29	0.64	0
	LGL	28	0.136	0.233	0.415	0.864	821 (684–1025)	0.74	0.98	29	7.15	0.90	0.28
	SVN	29	0.162	0.185	0.125	0.798	385 (353–423)	0.73	0.92	27	3.69	0.85	0
Great Lakes (GL)	RBW	19	0.211	0.234	0.101	0.851	1327 (940–2256)	0.70	0.96	18	5.02	0.78	0.06
	SPN	30	0.183	0.213	0.142	0.840	296 (279–316)	0.70	0.96	17	6.09	0.88	0.35
	GIL	28	0.185	0.230	0.198	0.840	147 (142–152)	0.72	0.98	26	6.76	0.81	0.38
	GRN	29	0.190	0.262	0.274	0.916	274 (259–290)	0.70	1	18	6.63	0.94	0.50
	ELK	20	0.139	0.256	0.460	0.871	282 (255–316)	0.71	0.96	18	6.38	0.83	0.28
	OKA	28	0.103	0.218	0.525	0.834	355 (317–402)	0.68	0.92	27	6.01	0.93	0.07
	GNV	30	0.147	0.207	0.289	0.845	391 (358–431)	0.67	0.98	27	9.15	0.96	0.33
	HOW	15	0.146	0.163	0.103	0.733	Inf (Inf‐Inf)	0.68	0.87	13	6.50	0.92	0.15
	EVE	7	0.057	0.136	0.582	0.646	Inf (Inf‐Inf)	0.66	0.80	7	6.13	0.86	0.57
	CRD	22	0.164	0.192	0.145	0.800	47 (46–48)	0.67	0.91	11	5.52	0.91	0.45

Note

*N* is the number of successfully genotyped individuals (Rapture and MHC), *H*_o_/*H*_e_ is observed and expected heterozygosity, respectively; *G*_IS_ is an *F*_IS_‐analogue inbreeding coefficient; Prop_poly_ for the Rapture data is the proportion of total polymorphic loci found in the populations; *N*_e_ is effective population size (with corresponding confidence intervals, CI); AF_del mut_ is the average allele frequency of deleterious alleles; Prop_del mut_ is the proportion of all deleterious mutations identified in the study that are found in a given population; N_haps_ is the average number of MHC haplotypes in each population; Prop_poly_ for the MHC data is the proportion of individuals in each population that contain more than one allele; and Prop_>2 alleles_ is the proportion of individuals in a population with more than two MHC alleles.

**FIGURE 2:**
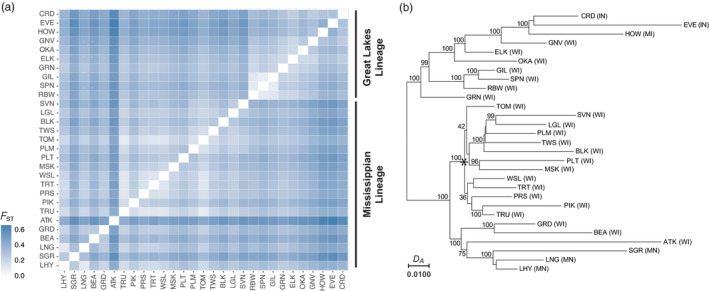
A heatmap of pairwise genetic difference among lakes (a) and neighbor‐joining tree indicating the presence of two lineages across our sampling region (b). Two clades in our Mississippian lineage (root marked with *) almost perfectly associated with the Wisconsin and Chippewa River basins. Bootstrap support values are labeled on major branches

Differentiation among populations was high (average *F*_ST_: 0.361, Figure 2a; Table S1). The lowest *F*_ST_ was measured between two Wisconsin populations found within 10 km of one another (SPN and GIL), and *F*_ST_ was comparatively low between all three proximal populations in this region (RBW, SPN, GIL, *F*_ST_: 0.076–0.105). This pattern was uncommon across the rest of the sampled populations. For example, many of the populations in northern Wisconsin were tightly clustered and highly differentiated, such as Pallette (PLT) and White Sand (WSL) lake, which are <2 km apart and had an *F*_ST_ of 0.239 and Plum (PLM) and Big Muskellunge (MSK) lake, which are <8 km apart and had an *F*_ST_ of 0.201. The highest *F*_ST_ was measured between Atkins Lake (ATK) in northern Wisconsin and EVE in Indiana (*F*_ST_: 0.661); however, overall ATK was more differentiated from other populations than any other lake (Figure 2a, average *F*_ST_ between ATK and other lakes: 0.511). Since ATK also contained a significantly larger number of samples (*n* = 54) than other sites, we randomly subsampled 30 individuals from ATK and regenerated pairwise *F*_ST_ values to test whether sample size could be driving the higher levels of differentiation. After reducing the sample size to 30, ATK still maintained the highest average pairwise differentiation with other sites indicating this pattern is not driven by sample size effects (average *F*_ST_ between reduced ATK and other lakes: 0.490; see Table S2). Outside of ATK, the largest pairwise *F*_ST_ value was measured between Sugar Lake in Minnesota (SGR) and EVE in Indiana (*F*_ST_: 0.575).

Patterns of differentiation across populations indicated sampling occurred across a biogeographic break (Figure 2a), and the population tree indicated two major genetic lineages (Figure 2b) across the sample region. Lakes in Minnesota and northern Wisconsin belonged to one clade, and the lakes in southeastern Wisconsin, Indiana, and Michigan belonged to the other [termed here the Mississippian (MS) and Great Lakes (GL) lineages]. We named these lineages to reflect current Great Lakes and Mississippi River drainage boundaries. Notably, two subclades within the MS lineage almost perfectly sort populations into the Chippewa and Wisconsin River basins (see top right inset in Figure 1 and the * in Figure 2b), with the exception of Pallette (PLT) and Big Muskellunge (MSK) lake, which are in the Chippewa River basin near the border of the Wisconsin River basin but aligned with the Wisconsin River basin populations.

**FIGURE 3:**
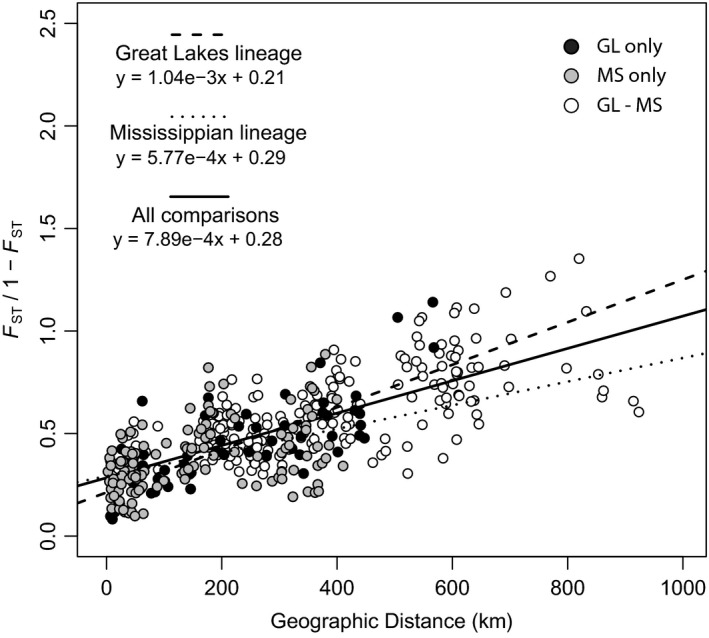
Isolation‐by‐distance graph. Genetic differentiation values (*F*_ST_/(1–*F*_ST_)) were generated with Rapture data. Within or among lineage pairwise comparisons are indicated by the color of fill—black circles are comparisons within the Great Lakes (GL) lineage, gray circles are comparisons within the Mississippian (MS) lineage, and white circles are comparisons between lineages. Pairwise comparisons with ATK, a highly differentiated population in the MS lineage, were not included in this plot

Mantel and partial Mantel tests indicated significant correlation between *F*_ST_/(1‐ *F*_ST_) and geographic distance (Figure 3; Table S3). A Mantel test with all sites was significant (*r *= 0.588, *p *< 0.001), but likely biased by hierarchical structure between the two lineages. Mantel tests restricted to populations within lineages showed significant IBD) in the GL lineage (*r *= 0.729, *p* < 0.001), but nonsignificant IBD in the MS lineage (*r* = 0.282, *p* = 0.099). When ATK was removed, the test for IBD in the MS lineage was significant (*r* = 0.466, *p* = 0.009), indicating that populations in close proximity to ATK had reduced the strength of the IBD relationship. Partial Mantel tests with ATK removed suggested lineage and geographic distance had a significant correlation to *F*_ST_/1 − *F*_ST_, but that lineage‐corrected geographic distance was a larger factor (*r* = 0.596, *p *< 0.001) than distance corrected‐lineage membership (*r* = 0.190, *p* = 0.003). Similarly, an AMOVA indicated that lineages accounted for 13.6% of the variance in the data (*p *< 0.001), whereas populations accounted for 25.6% of the variance (*p *< 0.009, Table 3). Given the lineage effects on our estimates of genetic differentiation, we limited our exploration of the relationships of variance in genetic differentiation to variance in population size [as *di* of log (surface area)] and average pairwise differentiation to log (surface area) to the MS lineage with ATK excluded (a total of 18 lakes, see Figure S1). We found no significant correlation between *F*_ST_/(1 − *F*_ST_) and *di* of log (surface area) (adjusted *r* ^2^ < 0.01, *p* = 0.615); however, the average pairwise differentiation for a lake was slightly negatively correlated with log (surface area) (adjusted *r* ^2^ = 0.22, *p* = 0.027).

**TABLE 3:** Analysis of molecular variance (AMOVA) with Rapture loci in 29 inland lake cisco populations

Source of variation	% variance	*F* statistic	*p*‐Values
Within individuals	0.4860	*F*_IT_	—
Among individuals in lakes	0.1223	*F*_IS_	<0.001
Among lakes in lineages	0.2557	*F*_SC_	<0.001
Among lineages	0.1358	*F*_CT_	<0.001

Observed and expected heterozygosity (*H*_o,_ *H*_e_) varied substantially (*H*_O_: 0.057–0.257, *H*_e_: 0.125–0.264) with the lowest *H*_o_ measured in Eve Lake in Indiana (EVE) and the highest in Trude Lake in northern Wisconsin (TRU). Estimates of inbreeding were similarly broad (*G*_IS_: 0.028–0.582) with the same two lakes accounting for the extremes (TRU: 0.028, EVE: 0.582). Estimates of effective population size (*N*_e_) were highly variable across populations (36–1781, Table 2). Median *N*_e_ across populations was 296 with most populations exhibiting an *N*_e_ between 100 and 1000 (*n* = 22). The two populations that had an *N*_e_ > 1000—White Sand (WSL) and Rainbow (RBW) lake—were both in Wisconsin, and the three lakes that had an *N*_e_ < 100—Sugar Lake in Minnesota (SGR), Black Oak Lake in northern Wisconsin (BLK), and Crooked Lake in Indiana (CRD)—were distributed across the sample range. An additional two populations generated ‘infinite’ estimates, EVE in Indiana and Howard Lake (HOW) in Michigan. An ‘infinite’ *N*_e_ is more likely a sign that the sample sizes in these populations were too small to generate a finite estimate with the LDNE method rather than a reflection of extremely large population sizes (see Do et al., 2014; Waples & Do, 2010). Due to potential sample size effects on estimates of *N*_e_ and the inability to generate bounded estimates for all sample sites, we did not include *N*_e_ in the genetic variables used in correlations.

### 3.2 Identifying putatively deleterious mutations

A total of 912 of the 7546 loci were successfully aligned to protein sequences from the Atlantic salmon genome. Of these 912 loci, 315 had alleles that coded for different protein sequences (i.e., nonsynonymous mutations), and 173 of these mutations (54.9%) were predicted to be putatively deleterious (PROVEAN score ≤ −2.5). The proportion of all identified deleterious mutations found in each population (i.e., with frequency >0 in a given population) averaged 0.95 and varied from 0.80 to 1 (Table 2), and the mean frequencies of deleterious mutations in each population averaged 0.71 and ranged from 0.66 to 0.74. Deleterious mutation metrics were similar between lineages, with an average frequency of deleterious mutations of 0.72 in the MS lineage and 0.69 in the GL lineage and an average ratio of deleterious mutations of 0.96 in the MS lineage and 0.93 in the GL lineage.

### 3.3 Haplotype diversity in the MHC

A total of 629 fish were successfully genotyped for MHC IIβ exon 2 (Table 2). High sequencing coverage across individuals was achieved, with an average coverage of 205 (standard deviation 123). Using these data, 73 haplotypes were discovered with >1 occurrence. A *Z*‐test of selection investigating the DN/DS ratio across all haplotypes indicated that DN >> DS (*p*‐value < 0.001, test statistic =3.81), providing strong evidence that MHC haplotypes in this system have historically undergone balancing selection. Most individuals in the dataset contained at least two MHC haplotypes (82% on average across populations, Table 2), and an average of 28% of individuals across populations contained >2 haplotypes. The rarefied number of haplotypes varied substantially, with estimates from 2.68 in Pike Lake (PIK) to 9.15 in Lake Geneva (GNV, average = 5.93). MHC haplotype frequencies did not closely reflect geographic proximity; large differences in MHC haplotypes were observed at small spatial scales (Figure S2). This was especially apparent for geographically proximate populations in northern Wisconsin. For example, Big Muskellunge (MSK) and White Sand (WSL) lakes had an almost completely different set of haplotypes despite being located <10 km apart. MHC haplotypes were also decoupled from underlying population genomic structure; for instance, the seq00111 and seq00024 haplotypes were found in high frequencies in populations from both major lineages (see next section, Data S1 and S2).

### 3.4 Relationships among genetic variables and spatial and environmental parameters

**FIGURE 4:**
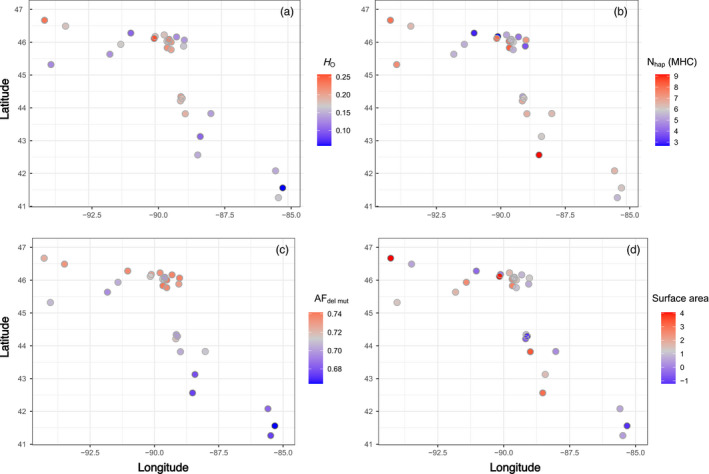
Visualization of three genetic variables (*H*_o_, *N*_hap_ (MHC), AF_del mut_), and one environmental variable (lake size) across our study area. Abbreviations: observed heterozygosity (*H*_o_), number of MHC haplotypes per population [*N*_hap_ (MHC)], the average allele frequency of putatively deleterious mutations in each population (AF_del mut_), and the surface area of each lake. Surface area is km^2^ and was log transformed to facilitate visualization

Sampled populations varied substantially in habitat metrics (Table 1; Table S4). For example, lake maximum depth varied from 13 to 72 m and lake size varied from 0.3 to 58 km^2^. Simple visualizations of genetic and environmental variables revealed that variation was not strictly partitioned by geography (Figure 4). For example, populations in northern Wisconsin near latitude 46° and longitude −90° had substantial variation in size, depth, and genetic metrics (Figure 4, Tables 1 and 2). Predictors chosen for initial screening included latitude, surface area, maximum depth, COSD (average and max), minimum oxythermal habitat thickness (average and min), minimum oxythermal habitat volume (average and min), and TDO3_30day_/TDO3_7day_/TDO3_max_ (average and max). Initial correlations indicated that different variants of the same model parameters were highly correlated, for example, the six TDO3 variables had pairwise correlation coefficients ranging from 0.91–1.00 (average *r* = 0.95). Of the six remaining variables after iterative removal with a threshold of VIF <5 ‐ latitude, surface area, depth, COSD(max), minimum oxythermal habitat volume(average), and TDO3_max_(max)—two pairs were correlated above the threshold of |*r*| >0.70 (depth and minimum oxythermal habitat volume, *r* > 0.86; TDO3_max_ and COSD, *r* > 0.80). Of these, we arbitrarily excluded minimum oxythermal habitat volume and COSD from the final list of predictors, but we treat interpretations of the remaining variables as potentially supporting for discarded variables.

**FIGURE 5:**
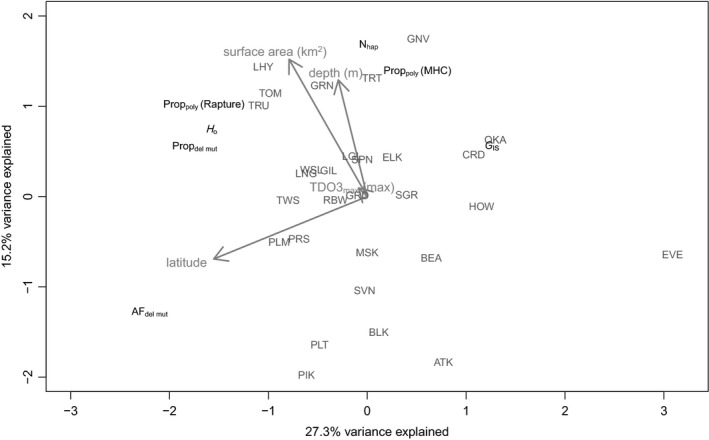
Redundancy analysis (RDA) investigating the influence of spatial and environmental variables (maximum depth, lake surface area, latitude, and TDO3) on genetic data [*H*_O_, Prop_poly_ (Rapture), *G*_IS_, AF_del mut_, Prop_del mut_, *N*_hap_, and Prop_poly_ (MHC)] for each population. Abbreviations for Rapture‐based metrics: observed heterozygosity (*H*_O_), proportion of total polymorphic alleles found in the populations [Prop_poly_ (Rapture)], inbreeding coefficient (*G*_IS_), the average allele frequency of putatively deleterious mutations in each population (AF_del mut_), and the proportion of deleterious mutations found in a population (Prop_del mut_). Abbreviations for MHC‐based metrics: number of MHC haplotypes per population (*N*_hap_), and proportion of individuals with >1 MHC haplotype [Prop_poly_ (MHC)]. Population abbreviations are in red and are identical to those described in Table 1. Latitude and surface area significantly influenced genetic variables according to an analysis of variance (*p* < 0.01, Table 4). Other environmental variables had *p*‐values > 0.05

Results from the RDA (global *p* < 0.001, 46% of variation in genetic metrics explained by predictor variables, *R* ^2^_adj_=0.375) demonstrated that AF_del mut_ and *G*_IS_ responded differently to predictors and were not strongly associated with other genetic variables, as compared to *H*_o_, Prop_del mut_, and Prop_poly_ (Rapture), which had similar loadings on RDA1&2 (Figure 5). MHC Prop_poly_ and *N*_hap_ also displayed similar loadings. Latitude contributed most to differences in genetic variables with high loadings on RDA1 (particularly AF_del mut_ and *G*_IS_), whereas lake surface area and maximum depth were associated with differences in genetic variables with high loadings on RDA2. Populations did not cluster tightly by geography indicating that variation in genetic and environmental variables is at least somewhat spatially decoupled. Analysis of variance on the RDA results indicated that latitude and surface area significantly influenced genetic variables (*p* < 0.01, Table 4). Depth nearly met the threshold for significance (*p* = 0.053). To assess whether previously removed environmental variables that were correlated with depth or TDO3_max_ (*r* > 0.80) might explain a significant portion of genomic variance, we replaced these variables and reran the ANOVA. When depth was replaced with minimum oxythermal habitat volume (average), an even lower proportion of variance was explained (*p* = 0.549). Similarly, when TDO3_max_ was replaced with COSD, the amount of genomic variance explained remained nonsignificant (*p* = 0.591).

**TABLE 4:** Variance inflation factors (VIF) and results from analysis of variance (ANOVA) on redundancy analysis (RDA) to assess which environmental variables significantly influenced genetic variables

Environmental variable	VIF	Variance	*F*	*p*‐value
Latitude	1.08	1.73	11.10	**0.001**
Surface area (km^2^)	1.61	1.02	6.56	**0.002**
Depth (m)	1.50	0.38	2.42	0.053
TDO3_max_ (max)	1.44	0.11	0.73	0.538

Note

ANOVA *p*‐values < 0.05 are in bold. See Figure 5 for RDA visualization.

**FIGURE 6:**
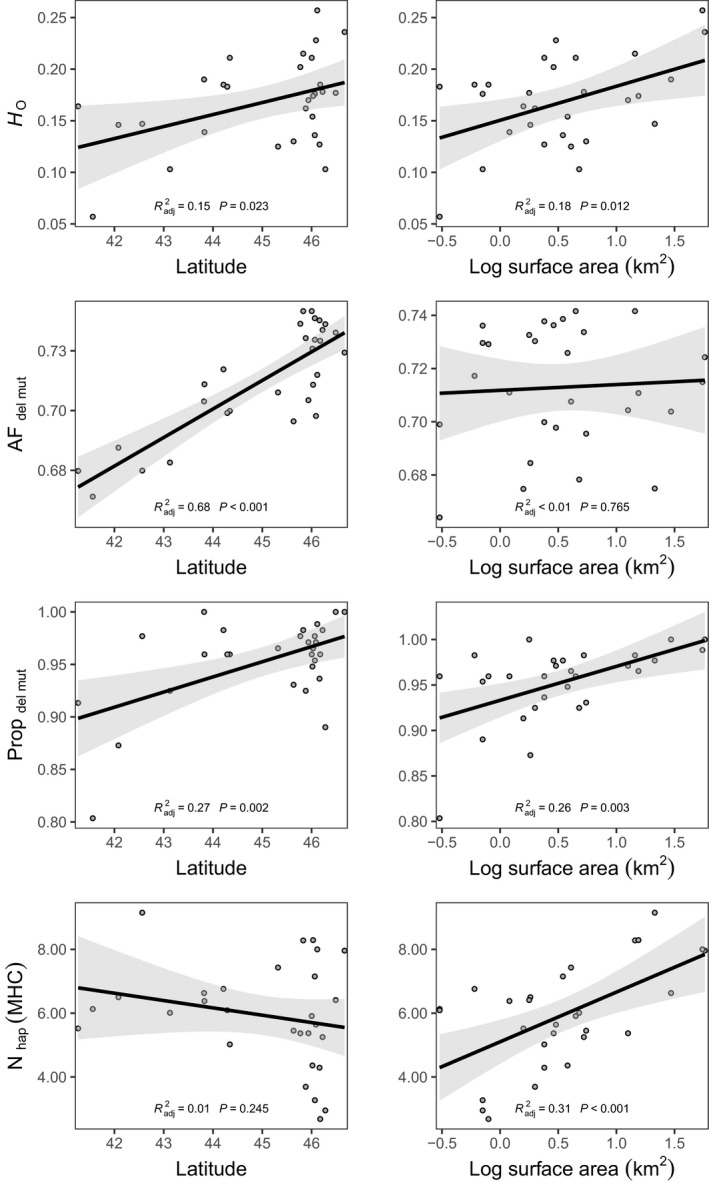
Relationships among four genetic metrics and latitude and lake surface area which were found to significantly influence genetic data in the redundancy analysis (Figure 5). Genetic metrics presented here include observed heterozygosity (*H*_O_), the average allele frequency of putatively deleterious mutations in each population (AF_del mut_), the proportion of deleterious mutations found in a population (Prop_del mut_), and the number of MHC haplotypes per population (*N*_hap_). *p*‐Values and correlation coefficients for each regression are denoted

Multiple linear regressions conducted with seven genetic metrics (Rapture data: *H*_o_, *G*_IS_, Prop_poly_, AF_del mut_, Prop_del mut_; MHC data: Prop_poly_, *N*_hap_) as the response variables and four spatial or environmental metrics (latitude, surface area, depth, TDO3_max_) as the predictor variables indicated that the predictor with the highest relative importance on average was latitude followed by surface area, and maximum depth (Table 5). However, the relative importance of different predictors varied substantially across regressions, with surface area explaining the largest proportion of variance in *H_o_*_,_ Prop_poly_ (Rapture), and *N*_hap_ (MHC), and latitude explaining the most variance in *G*_IS_, AF_del mut_, Prop_del mut_, and Prop_poly_ (MHC). The average amount of variation explained in each regression was 0.38, with the lowest value (0.18) found for Prop_poly_ (MHC) and the highest (0.68) for AF_del mut_. Univariate regression with the significant predictor variables in the RDA (latitude and surface area) revealed significant positive relationships between latitude and *H*_o_, AF_del mut_, and Prop_del mut_, and significant positive relationships between surface area (log transformed to aid visualization) and *H*_o_, Prop_del mut_, and *N*_haps_ (Figure 6). *H*_o_ and Prop_del mut_ were significantly and positively correlated with latitude and surface area, while the other two genetic variables (AF_del mut_ and *N*_hap_) were significantly correlated for one metric, but not the other. Notably, AF_del mut_ was not significantly correlated with surface area but was highly correlated with latitude (highest *r* ^2^ and lowest p‐value of all eight regressions).

**TABLE 5:** Results from multiple linear regressions investigating the relationships between genetic and environmental variables

				Relative importance			
Dataset	Genetic variable	*r* ^2^	*p*‐value	Latitude	Surface Area (km^2^)	Depth (m)	TDO3_max_
Rapture	*H*_O_	0.33	**0.008**	0.35	0.57	0.04	0.04
	Prop_poly_	0.42	**0.002**	0.30	0.35	0.29	0.06
	*G*_IS_	0.21	**0.046**	0.56	0.14	0.24	0.06
	AF_del mut_	0.68	**1.51E‐06**	0.97	0.01	0.01	0.01
	Prop_del mut_	0.47	**5.27E‐04**	0.55	0.19	0.22	0.04
MHC	*N*_hap_	0.30	**0.013**	0.18	0.66	0.14	0.02
	Prop_poly_	0.17	0.071	0.48	0.29	0.21	0.02
	Average	0.38	**0.017**	0.48	0.31	0.17	0.03

Note

The relative importance of each variable in the regression was calculated using the lmg method implemented in the R package relaimpo. *p*‐Values <0.05 are in bold.

## 4 DISCUSSION

Our coupled genomic–environmental approach offers new insights into the factors influencing genetic diversity and differentiation at the southern range boundary for cisco. High levels of genetic differentiation between populations were punctuated by a phylogeographic break and residual patterns of isolation‐by‐distance (IBD). Although the prevalence of deleterious mutations and inbreeding coefficients were significantly correlated with latitude, largely neutral (Rapture) and non‐neutral (MHC) genetic diversity were most strongly correlated with lake surface area. Our results provide valuable information for guiding restoration efforts as well as spatial and environmental factors that may govern the persistence of ciscoes and other cold‐water specialist species as the climate continues to warm.

### 4.1 Historic vicariance and drift in small, isolated populations

The discovery of two divergent sets of populations in our study that we hypothesize are distinct genetic lineages provides new insights on the phylogeographic history of *C*. *artedi*. Turgeon and Bernatchez (2001a, 2003) first found evidence with microsatellites for two cisco lineages hypothesized to be associated with putative glacial refugia (see Eshenroder & Jacobson, 2020), and this phylogenetic break was recently detected again in reduced‐representation sequencing data (Piette‐Lauzière et al., 2019). Only a handful of populations in these studies were sampled across the region where the two lineages meet, and thus, the location of the transition from one lineage to the other has been placed in the general area of Lake Nipigon (Ontario, Canada) and Lake Superior. In our study, we found that what we identified as the MS lineage extends below the southern shore of Lake Superior across northern Wisconsin with the first inland populations associated with the GL lineage occurring in central and southeastern Wisconsin. While we hypothesize that our GL and MS lineages correspond to the two lineages identified in previous studies and believe that our data span a boundary between the lineages, it is important to note that our study covers a relatively small portion of the species range and our findings should be reevaluated in future studies that include additional populations. Within MS lineage cisco populations in northern Wisconsin, genetic clades were almost perfectly associated with the Chippewa and Wisconsin River basins. A similar pattern of genetic association within these river basins has also been found in walleye (*Sander vitreus*; Bootsma, Miller, et al., 2021), suggesting these drainages maintained corridors of migration between nearby refugia before receding water levels isolated inland lakes.

The drift that followed the isolation of inland lake populations is reflected in the high levels of genetic differentiation we found between our inland cisco populations as well as the pattern of increased average pairwise differentiation with decreased lake size in the MS lineage. These high levels of interpopulation differentiation are similar to those observed in other northern temperate lacustrine species including lake trout (*Salvelinus namaycush*) and brook trout (*Salvelinus fontinalis*) (Ferchaud et al., 2020; Perrier et al., 2017). In several cases, this high genetic differentiation was found across very small spatial scales. In the Chippewa River basin, White Sand (WSL), Pallette (PLT), Trout (TRT), and Big Muskellunge (MSK) lakes are all <5 km apart, and the level of pairwise differentiation suggests little or no gene flow has occurred among these lakes for thousands of years. Despite the high levels of interpopulation differentiation, a small but significant pattern of IBD was still present in both lineages when the effect of Atkins Lake (ATK) on the MS lineage was removed. ATK had the lowest genetic diversity and smallest proportion of total alleles in the MS lineage. This could indicate that a small population size in ATK is driving increased amounts of drift, differentiation, and inbreeding (Franklin, 1980); however, ATK did not exhibit a correspondingly elevated inbreeding coefficient as observed in two populations with equal or lower genetic diversity, Okauchee (OKA) and Eve (EVE). In light of this, it is worth considering that ATK is unique among our sampled lakes in that it contains the dwarf cisco ecotype. Dwarf coregonines are smaller‐bodied, spawn earlier, and typically exhibit shorter life spans than their alternative ecotype counterparts (Mann & McCart, 1981; Svardson, 1950). Transplanted dwarf cisco maintain their early spawning behavior (Shields & Underhill, 1993) suggesting heritable differences between ecotypes exist, and since contemporarily ATK is only known to contain the dwarf ecotype, this could lead to a small but significant increase in invariant loci when a panel developed to target polymorphic loci in alternative ecotype cisco is used.

### 4.2 Spatial and environmental correlates of genetic metrics

Loss of adaptive genetic variation and inbreeding depression via the accumulation of deleterious mutations are major threats to the persistence of small, isolated populations (Charlesworth & Willis, 2009; Hedrick & Garcia‐Dorado, 2016; Hedrick & Kalinowski, 2000; Keller & Waller, 2002; Lande, 1995). Therefore, we used a variety of metrics to quantify neutral (Rapture) and non‐neutral (MHC) genetic diversity, inbreeding, and the frequency of putative deleterious mutations in inland lake cisco populations at the southern extent of their range. Understanding how spatial or environmental factors are correlated with any of these risk factors can provide managers with potential avenues for mitigating problems before they lead to extirpation, as well as identify characteristics of suitable lakes for reintroductions.

Latitude and the frequency of putative deleterious mutations exhibited the strongest correlation between a predictor variable and a genetic metric in our data. Across our sampled lakes, the proportion and frequency of deleterious mutations decreased the further south a population was located. While the proportions of deleterious mutations across populations of inland cisco were similar in range to those found in North American lake trout populations in Ferchaud et al. (2018), the allele frequencies we measured appear much larger than those of widespread continuous populations where most deleterious mutations tend to be a minor allele (Conte et al., 2017; Zhang et al., 2016). The most similar systems to our small, isolated inland cisco populations that have had mutational load evaluated are North American lake trout and brook trout populations; however, differences in methodologies make comparisons challenging. For example, Ferchaud et al. (2018), Ferchaud et al. (2020) used the same PROVEAN threshold we did, but interpreted the minor allele as the deleterious mutation regardless of +/− state due to expectations that strongly deleterious mutations should be purged and in low frequency. The range of average deleterious mutation allele frequencies under this assumption for lake trout was 0.12–0.24 (Ferchaud et al., 2018), and when we analyze our data using the same assumption, our average deleterious mutation allele frequencies similarly range from 0.07 to 0.18, so it is possible that Ferchaud et al. (2018) may have also found high deleterious allele frequencies if they had followed the same interpretation we employ here. A PROVEAN score divides nonsynonymous mutations into binary categories of putatively neutral and putatively deleterious, but within identified putatively deleterious mutations there is no way to distinguish mildly deleterious mutations from strongly deleterious mutations. Our inland cisco populations display patterns indicating a strong influence of drift, which reduces the ability of selection to purge mutational load, especially mildly deleterious mutations, which do not have large fitness effects (Crow, 1993; Glemin, 2003; Kimura et al., 1963). The relatively high average allele frequencies of deleterious mutations we measured could be reflective of an accumulation of mildly deleterious mutations rather than high frequencies of strongly deleterious mutations.

Predicting what patterns of mutational load we might expect to see in inland cisco populations along the southern (trailing) edge of the species range is not straightforward. Several studies have observed increased mutational load on the edges of a species range (Henry et al., 2015; A. Perrier et al., 2020; Willi et al., 2018; Zhang et al., 2016) as a result of increasingly smaller peripheral populations driving increased genetic drift and less effective purifying selection (Crow, 1993; Kimura et al., 1963; Whitlock, 2000). Alternately, many studies have simulated or observed increased mutational load and decreased heterozygosity specifically at the leading edge of a species range as a result of the demographic processes associated with colonization (Lohmueller et al., 2008; Peischl et al., 2013; Rougemont et al., 2020). Our observations of increased heterozygosity and increased mutational load with latitude in southern inland cisco populations do not perfectly align with either of these previously observed patterns of mutational load. The majority of study systems that have been used to examine mutational load across species ranges thus far have exhibited at least some level of migration and interpopulation gene flow (e.g., Rougemont et al., 2020; Willi et al., 2018; Zhang et al., 2016), unlike cisco populations, which were successively isolated in lakes as glaciers receded over thousands of years (Dyke & Prest, 1987). The small but significant correlations for decreased heterozygosity and increased inbreeding we observed the further south a population was found could expose recessive deleterious mutations to selection and lead to purging rather than accumulating mutational load (Hedrick, 1994; Hedrick & Garcia‐Dorado, 2016; Kirkpatrick & Jarne, 2000; Lande & Schemske, 1985; Lynch et al., 1995). Perrier et al. (2017) investigated the proportion of deleterious mutations in isolated populations of lake trout in Quebec, Canada, and although they did not examine whether these proportions correlated with latitude, they similarly detected a weak but significant correlation between latitude and inbreeding, suggesting positive associations between latitude and the frequency of deleterious mutations may exist in other glacial lake populations. Ultimately, extended sampling across a larger portion of the species range is needed to assess the relative roles of drift and selection versus demographic processes such as directional colonization and lineage admixture on the latitudinal pattern of mutational load we observed in southern inland lake cisco populations.

Genetic diversity in inland cisco populations was also linked to lake‐specific environmental variation, reflecting both static and dynamic variables. The number of MHC haplotypes observed in each population, which showed no association with latitude, was positively correlated to lake surface area. No other patterns among populations or correlations with environmental factors were found in the MHC IIβ exon 2, including between lineages, suggesting that diversity in this gene may be linked to population size and drift. Some measures of diversity that were associated with broad latitudinal patterns were also correlated to lake surface area, including neutral diversity and the proportion of deleterious mutations in cisco populations. A positive relationship between genetic diversity and lake size has also been found in several other salmonids, including lake trout (Perrier et al., 2017; Valiquette et al., 2014) and brook trout (Castric et al., 2001), as well as three‐spined stickleback (*Gasterosteus aculeatus*) (Bolnick & Ballare, 2020; Caldera & Bolnick, 2008). Moreover, Bolnick and Ballare (2020) found that genome‐wide heterozygosity in lacustrine three‐spined stickleback was not correlated with resource diversity and morphological variation, consistent with the expectation that larger population sizes supported by larger lakes experience less drift and therefore increased neutral genetic diversity. Decreasing amounts of drift in larger effective population sizes has also been suspected of driving the positive correlation of proportion of deleterious mutations and lake size observed in lake trout (Perrier et al., 2017).

It is notable that estimates of TDO3 were not significantly correlated with any genetic variables, and this may reflect the complexities of using one‐dimensional modeling to describe three‐dimensional habitat. TDO3 and related metrics are useful, but these metrics and corresponding one‐dimensional modeling and deep‐hole lake monitoring cannot capture isolated pockets of refugia habitat that are often present in large lakes. For example, when using three‐dimensional thermal and oxygen models to simulate Lake Mendota, Wisconsin, results indicated that the majority of cisco habitat in late summer may actually be around the 12–15 m contour of the lake where internal seiche and downwelling events are more likely to occur, bringing pockets of oxygenated water into the hypolimnion, which may act as refugia habitat to sustain the remnant cisco population (see Figure S3). Similarly, by their nature, one‐dimensional models also cannot capture these pockets of refugia within large lakes with multiple deep locations and abrupt changes in bathymetry. In addition, TDO3 only captures one important lake characteristic influencing inland cisco populations. Lake size, which was correlated with genetic diversity, likely encompasses a multitude of factors influencing population size such as prey availability, refuge habitat from predators, and spawning habitat.

### 4.3 Conserving inland lake cisco populations at the southern extent of their range

Genomic data can provide valuable information to guide decision making in fish conservation (Bernos et al., 2020), and our results provide multiple useful inferences for conserving marginal cisco populations. Most obviously, knowing the extent of the two phylogeographic lineages as well as the relationships between deleterious mutations with latitude and diversity with lake surface area can help inform common freshwater conservation strategies including reintroduction of extirpated populations and the augmentation of diminished or imperiled populations through outcrossing (Anderson et al., 2014; Lamothe & Drake, 2019; Marsh et al., 2005; Weise et al., 2020). Lutz et al. (2021) found that using multiple, compatible source populations for reintroduction of endangered Macquarie perch (*Macquaria australasica*) benefitted survival and recruitment, and a similar approach may maximize successful reintroductions of inland lake cisco given the apparent influence of population size and drift over selection and adaptation in inland lake populations. In Wisconsin, where historic MS and GL lineages converge, careful consideration should be given before implementing a reintroduction or augmentation plan that involves mixing divergent genetic lineages as the presence of fixed differences in highly divergent populations is a risk factor for outbreeding depression (Frankham et al., 2011). A current reintroduction initiative involving live‐transfers of cisco from WSL to nearby Crystal and Sparkling lakes in fall 2020 will provide important baseline information for the use of regional single‐ and multiple‐source strategies more broadly.

Augmentation through supplementation is rare in inland cisco populations compared to other northern lacustrine salmonids such as lake whitefish (*Coregonus clupeaformis*) and lake trout, which are more likely to be targeted by recreational fishers (Frey, 1955). A handful of records from cisco stocking events in the early 20^th^ century in Indiana and Michigan exist (Frey, 1955; Homola et al., 2021), and increases in mitochondrial diversity from historic stocking in a few Michigan lakes has been observed (Homola et al., 2021). To the best of our knowledge, none of the lakes included in our study were ever stocked, and Homola et al. (2021) found no evidence of stocking in the one lake we sampled in Michigan (HOW). The broad patterns in IBD and frequency of deleterious mutations we observed across our sampled lakes further support a lack of evidence for stocking events. Contemporarily, conservation measures for inland lake cisco populations focus on mitigating cold‐water habitat degradation and controlling harvest (e.g., Jacobson et al., 2010, 2012; Tingley et al., 2019) rather than supplementation. With little empirical evidence available for the influences of supplementation on inland lake cisco, it is difficult to predict the viability of this strategy to retain beneficial genetic diversity. Genetic rescue of depleted populations through outcrossing has been shown to increase diversity and fitness in many small, isolated populations (Fitzpatrick et al., 2020; Frankham, 2015, 2016). There is some contention, however, on whether ideal source populations used for augmentation should maximize the genetic diversity of target populations (Ralls et al., 2020) or minimize the introduction of strongly deleterious mutations (Kyriazis et al., 2020; Robinson et al., 2020). Ferchaud et al. (2018) found increased neutral diversity and fewer deleterious mutations in stocked versus unstocked populations of lake trout, attributing this trend to a reduction of the influence of drift through supplementation. Given the pattern of decreased mutational load in southern inland lake cisco populations, however, the introduction of new deleterious mutations could be problematic. Additionally, supplementing inland lake cisco populations with fish from the Laurentian Great Lakes could be detrimental regardless of mutational load since these populations have been observed to contain different phenotypic and behavioral adaptations from native inland lake populations (Jacobson et al., 2018), which are also risk factors for outbreeding depression (Frankham et al., 2011). Given the unpredictability of augmentation, it may be best to document interpopulation interactions through cisco reintroduction initiatives before applying this approach to vulnerable extant populations.

The correlation we observed between genetic diversity and lake surface area suggests one key to resilience in southern inland lake cisco populations may be maintaining populations that are large and diverse enough to survive the stochastic demographic, environmental, or genetic factors that drive the extinction of small populations (Frankham, 2008; Pimm, 1991). The predictor variables examined for correlations to genetic metrics in our study are only a subset of the landscape heterogeneity predicted to play a role in the persistence of inland lake cisco populations. While larger lake size was found to be a good predictor of the presence of historic cisco populations in Indiana (Honsey et al., 2016), non‐point‐source nutrient loading, urbanization, and sedimentation from land‐use are all observed or predicted to influence the quality of contemporary cisco habitat in southern lakes (Honsey et al., 2016; Jacobson et al., 2012; Magee et al., 2019). Additional threats are lake‐specific, such as the introduction of invasive species like rainbow smelt *Osmerus mordax* (Hrabik et al., 1998). The combined interaction of these idiosyncratic factors with fine‐scale heterogeneity in genetic diversity likely explains the lack of a predictable pattern to extirpations (Lyons et al., 2015; Renik et al., 2020). Current recommendations for protecting suitable cold‐water habitat suggest management at the catchment scale (Jacobson et al., 2013) jointly with adapted regional plans to address threats such as climate change (Tingley et al., 2019), and our findings of broad, latitudinal, and local‐scale patterns of maladaptation and genetic diversity support this approach for conserving southern inland lake cisco populations. The mosaic pattern of observed extirpations in this region coupled with the high levels of differentiation we observed at small spatial scales and correlations of lake size and diversity suggest that genetic resilience in inland lake cisco populations is closely associated to levels of genetic drift. The most successful conservation strategies, therefore, may be those that adequately account for the fine‐scale environmental processes that influence population sizes in order to reduce the chance of stochastic natural or anthropogenic events resulting in further extirpations.

## CONFLICT OF INTEREST

The authors declare no conflict of interest.

## Supporting information

Table S1

Table S2

Table S3

Figure S1

Figure S2

Data S1

Data S2

Table S4

Figure S3

## Data Availability

Sequence data for the 129 individuals used in the development of the Rapture panel were previously uploaded to the National Center for Biotechnology Information (NCBI) sequence read archive (BioProject: PRJNA557717). Rapture sequence data used in analyses in this manuscript were georeferenced in the Genomic Observatories MetaDatabase (https://n2t.net/ark:21547/DwZ2) and uploaded to the NCBI sequence read archive (BioProject: PRJNA771945). Scripts and supplemental material for the hydrodynamic model will be uploaded to Dryad upon acceptance.
