# Corrigendum

**DOI:** 10.1111/eva.13342

**Published:** 2022-01-26

In the article by Ackiss et al., the authors would like to update the Dryad information.

In the current Data Availability Statement, the last sentence reads:

“Scripts and supplemental material for the hydrodynamic model will be uploaded to Dryad upon acceptance.”

This should read:

“Scripts and supplemental material for the hydrodynamic model are available on Dryad: https://datadryad.org/stash/dataset/doi:10.5061/dryad.tx95x69zw”.
