# Supplementary material for: Genomic and environmental influences on resilience in a cold‐water fish near the edge of its range
Source: Evol Appl. 2021 Nov 9;14(12):2794–814. doi: 10.1111/eva.13313 (PMC8674893; doi:10.1111/eva.13313)
Supplement: Supplementary file 4 — Figure S1 [file EVA-14-2794-s008.doc]

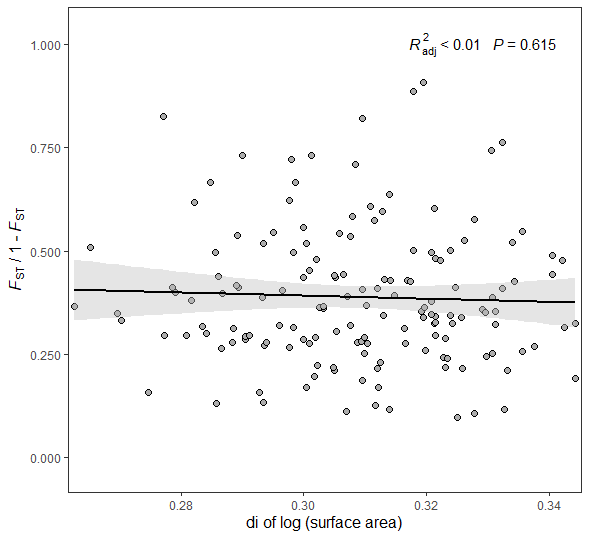


**A**

**
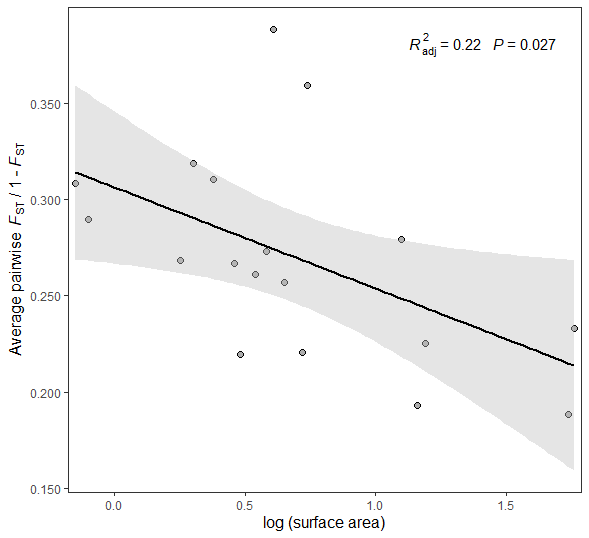
**

**B**

**Supplemental File S4.** The relationships between (A) genetic differentiation (*F*_ST_/1- *F*_ST_) and Prunier et al.’s (2017) *di* of log (surface area) and (B) average pairwise *F*_ST_/1- *F*_ST_ and log (surface area) in our Mississippian sites.
