# Supplementary figures and images for: Genomic and environmental influences on resilience in a cold‐water fish near the edge of its range
Source: Evol Appl. 2021 Nov 9;14(12):2794–814. doi: 10.1111/eva.13313 (PMC8674893; doi:10.1111/eva.13313)

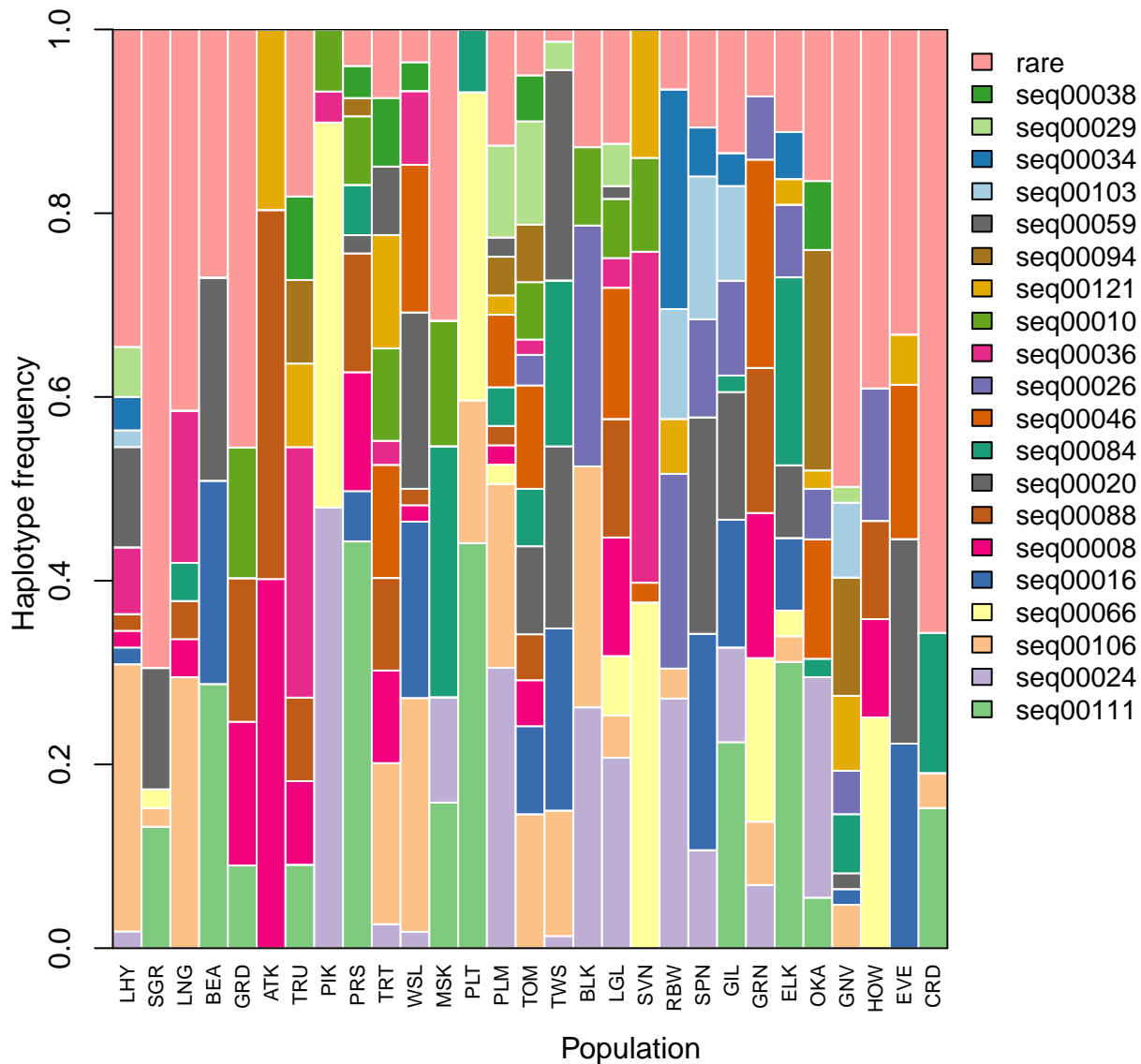

Supplement: Supplementary file 5 — Figure S2 [file EVA-14-2794-s001.pdf]
