# Supplementary material for: Genomic and environmental influences on resilience in a cold‐water fish near the edge of its range
Source: Evol Appl. 2021 Nov 9;14(12):2794–814. doi: 10.1111/eva.13313 (PMC8674893; doi:10.1111/eva.13313)
Supplement: Supplementary file 9 — Figure S3 [file EVA-14-2794-s003.docx]

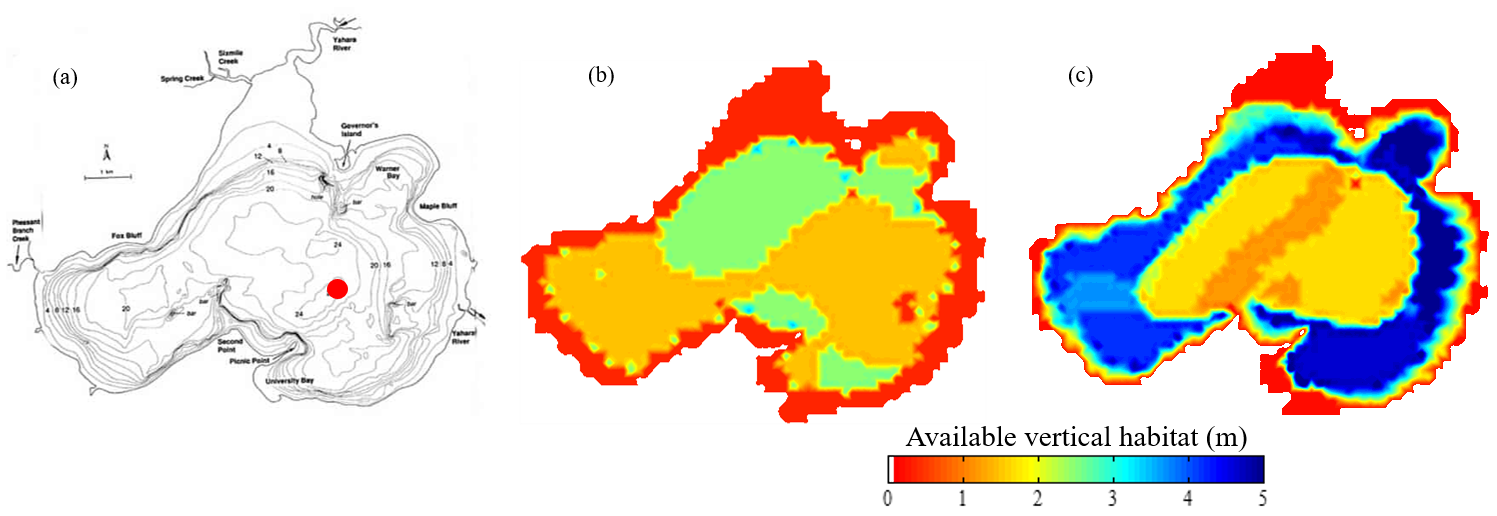


Three dimensional oxythermal habitat modeling results for Lake Mendota in Madison, WI, USA. Model used simulated temperature and dissolved oxygen in three dimensions for Lake Mendota during representative ‘typical’ meteorological conditions of air temperature and wind speed. (a) shows bathymetric map for the lake, with the deep hole location identified with the red circle. (b) show available vertical oxythermal habitat in meters for mid-August. (c) shows available vertical oxythermal habitat in meters for mid-September. Modeling results show that in both August and September, typically the periods with limited oxythermal habitat in this lake, less habitat is available in the deepest portions of the lake, compared to areas where the depth is slightly shallower, ranging from 16-20m. Data provided by author Madeline Magee.
